# Cocaine reward and reinstatement in adolescent versus adult rodents

**DOI:** 10.3389/fnbeh.2023.1278263

**Published:** 2024-01-05

**Authors:** Amy A. Arguello, Christian T. Valade, Luciano S. Voutour, Christopher A. Reeves

**Affiliations:** Department of Psychology, Interdisciplinary Science and Technology Building, Michigan State University, East Lansing, MI, United States

**Keywords:** adolescence, context, self-administration, cocaine seeking, reinstatement, cocaine conditioned place preference, incubation, cue

## Abstract

Adolescence is a critical juncture when initiation of drug use intersects with profound developmental changes in the brain. Adolescent drug use increases the risk to develop substance use disorders (SUDs) later in life, but the mechanisms that confer this vulnerability are not understood. SUDs are defined by cycles of use, abstinence, and relapse. Intense craving during drug-free periods is often triggered by cues and environmental contexts associated with previous use. In contrast to our understanding of stimuli that elicit craving and relapse in adults, the behavioral processes that occur during periods of abstinence and relapse in adolescents are poorly understood. The current mini-review will summarize findings from preclinical rodent studies that used cocaine conditioned place preference and operant cocaine self-administration to examine subsequent effects on reward, relapse and incubation of craving.

## Introduction

Adolescence is a critical period of development when brain maturation intersects with initiation of drug-taking behavior (Chambers et al., 2003; Stanis and Andersen, 2014; Caballero et al., 2016). Drug use during adolescence increases the risk to develop substance use disorders (SUDs), with the severity of diagnosis at adolescence related to more pronounced negative outcomes later in life (Wagner and Anthony, 2002; Ramo et al., 2012; Winters et al., 2014; McCabe et al., 2022; Volkow and Wargo, 2022). Adults diagnosed with SUDs suffer from chronic relapse often after exposure to drug use environments (contexts), paraphernalia (cues), or stress (Ehrman et al., 1992; Childress et al., 1999; Foltin et al., 2000; Saunders, 2017). Increased craving for drug, or incubation of craving, despite long periods of abstinence poses a particularly difficult challenge for relapse prevention and the successful treatment of SUDs (Gawin and Kleber, 1986; Grant et al., 1996; Garavan et al., 2000; Parvaz et al., 2016). In contrast to our understanding of craving and relapse in adults, the behavioral processes during periods of abstinence and relapse in adolescents are poorly understood.

Human adolescents have distinct patterns of drug use, with longer periods of abstinence between bouts of use compared to adults (Winters et al., 2014; Silvers et al., 2019; Moska et al., 2021). Up to 86% of adolescents relapse after leaving treatment centers, suggesting that the abstinence period is also a critical window of risk for adolescents (Acri et al., 2012; Squeglia et al., 2019; Moska et al., 2021). In regard to cocaine use, existing studies have primarily focused on problematic adult use, cue reactivity, craving and relapse. However, from 2012 to 2018 cocaine-related mortality in adolescent populations increased three-fold. FDA-approved treatments for cocaine use disorders for adults and adolescents are lacking and therefore problematic use during adolescence has the potential to remain untreated and persist into adulthood (LaBossier and Hadland, 2022; McCabe et al., 2023).

Several questions about adolescent drug use, relapse and abstinence remain: what types of drug-associated stimuli trigger craving and relapse in adolescent populations? When is craving highest during abstinence? Are adolescents more sensitive to particular relapse triggers compared to adults? Use of preclinical rodent models of reward and relapse are necessary for a comprehensive understanding of adolescent drug-related behaviors, which is critical to tailor existing behavioral interventions to adolescents and develop new prevention strategies for this vulnerable age group. The current mini-review will summarize findings from preclinical rodent studies that used cocaine conditioned place preference and operant cocaine self-administration to examine subsequent effects on reward, relapse and incubation of craving. We refer the reader to excellent reviews on the behavioral effects of adolescent nicotine, alcohol, cannabis and other stimulant use and the effects of acute or passive adolescent cocaine exposure (Barron et al., 2005; O’Dell, 2009; Marco et al., 2011; Spear, 2016; Walker et al., 2017; Stringfield and Torregrossa, 2021).

### Cocaine conditioned place preference (cocaine-CPP) in adolescent and adult rats

Cocaine CPP is a preclinical rodent model used to evaluate the rewarding effects of drug by pairing experimenter-delivered infusions of cocaine in a specific context (Carlezon, 2003; Prus et al., 2009). In general, an initial preference test occurs in a two or three compartment apparatus to assess baseline preferences for a specific compartment. Cocaine is paired in the least preferred compartment in biased designs or randomly assigned to a compartment in unbiased designs. During conditioning trials (15–60 min), rats receive an intraperitoneal (i.p.) injection of saline and are then placed into one compartment (saline-paired). The next day, or 4–5 h later, rats receive an injection of cocaine and are then placed into the second compartment (cocaine-paired). Alternate pairings of cocaine and saline occur 1-2x/day over 2–5 days. On the test day, rodents are placed back in the CPP apparatus (center, for 3 compartment setup) and allowed to freely explore all compartments. Preference for the cocaine-paired side can be measured in several ways (1) ratio of time spent in the cocaine-paired side/ total time in both sides, (2) difference in time spent in the cocaine- vs. saline-paired side or (3) difference in time spent in the cocaine-paired side during pre- vs. post-conditioning. More time spent in the cocaine-paired side suggests that CPP for the cocaine-associated context was formed (Prus et al., 2009; Olmstead, 2011).

**Figure 1:**
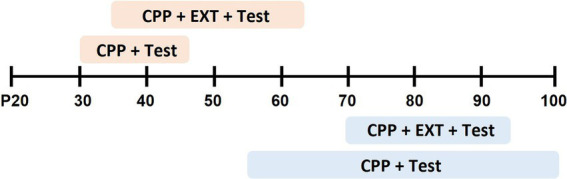
Summary of age range in experiments that employed cocaine conditioned place preference training and test (CPP + Test) or cocaine CPP, extinction training and Test (CPP + EXT + Test). Shaded boxes indicate the length of behavior across postnatal days (P) for adolescent (orange) or adult (blue) rats.

Studies that examined age-dependent differences in cocaine reward began behavioral training for adolescent rats at an average post-pubertal age of postnatal day 36 (P36, range from P30–41) and for adults at P72 (range from P55–102) (Figure 1). Studies in the current review delivered cocaine at doses between 3 and 40 mg/kg and used either biased or unbiased designs. Three types of CPP tests were utilized: (1) CPP tests with no cocaine onboard to examine the rewarding property of the cocaine-paired context, (2) CPP re-tests with no cocaine onboard to examine extinction of CPP- rats freely explore all compartments during unpaired extinction training or are confined to the former saline- or cocaine-paired sides during explicit paired extinction, or (3) cocaine-primed CPP in which cocaine (5 or 10 mg/kg) was administered before the CPP test (Table 1).

**Table 1:** Summary of key studies that compared adolescent and adult cocaine conditioned place preference (CPP).

Author	Rat Strain, Sex & Housing	Behavioral Range			CPP Training	EXT Training	Test	Adol < Adult	Adol = Adult	Adol > Adult
		Juv/Early	Adol	Adult						
Campbell et al. (2000)	SD, ♂ ♀ Bred in house Single at train		P32 - 36	P65/78 - 69/82	Biased 5, 10 mg/kg 3d, 2x/d, 15min	N/A	**CPP**: 15min		**CPP**: 5, 10 mg/kg	
Badanich et al. (2006)	SD, ♂ Bred in house Grouped	P30 - 35	P40 - 45	P55 - 60	Biased Pref Test: 15min 5, 20 mg/kg 4d, 2x/d, 15min	N/A	**CPP**: 15min		**CPP**: 20 mg/kg Early/ late adol = adult **CPP**: 5mg/kg Early > adol = adult	
Brenhouse et al. (2008)	SD, ♂ Housing N.S.	P24 - 27	P41 - 44	P102 - 105	Unbiased Pref Test: 30min 10, 20, 40 mg/kg 2d, 2x/d, 60min	N/A	**CPP**: 30min		**CPP**: 40 mg/kg Juv = adol = adult	**CPP**: 10, 20 mg/kg Adol > juv, adult
Zakharova et al. (2009a)	SD, ♂ Paired		P34 - 38	P66 - 70	Biased Pref Test: 30min 3, 5, 7.5, 10, 20 mg/kg 3d, 2x/d, 30min	N/A	**CPP**: 30min	**CPP**: 10 mg/kg, ♂		**CPP**: 5, 7.5 mg/kg, ♂
Zakharova et al. (2009b)	SD, ♂ ♀ Paired		P34 - 38	P66 - 70	Biased Pref Test: 30min 1, 3, 5, 7.5, 10, 20 mg/kg 3d, 2x/d, 30min	N/A	**CPP**: 30min	**CPP**: 10 mg/kg, ♀♂	**No CPP**: 1 mg/kg, ♀ **CPP**: 3, 5 mg/kg, ♀	**CPP**: 5, 7.5 mg/kg, ♂
Brenhouse and Anderson (2008)	SD, ♂ Housing N.S.		P38 - 45	P77 - 81	Unbiased Pref Test: 30min 10, 20 mg/kg 2d, 2x/d, 60min	CPP re-test 1x/d, 30min until < 50% CPP	**CPP**: 30min **Coc-prime**: 5 mg/kg		**CPP**: 20 mg/kg **Coc-prime**: 20 mg/kg	**CPP**: 10 mg/kg **EXT**: # of sessions **Coc-prime**: 10 mg/kg
Brenhouse et al. (2010)	SD, ♂ Housing N.S.		P38 - 46/48	P75 - 83/85	Unbiased Pref Test: 30min 20 mg/kg 2d, 2x/d, 60min	Unpair: 30min (CPP re-test) Explicit Pair: 30min (saline each side)	**CPP**: 30min **Coc-prime**: 5 mg/kg	**Coc-prime**: explicit grp	**CPP**: 20 mg/kg **EXT**: explicit grp **Coc-prime**: unpair grp	**EXT**: unpair grp
Guerin et al. (2021)	SD, ♂ Single at train		P35 - 53/56 P35 - 60/63	P70 - 88/91	Random & Biased Pref Test: 20min Match dose to Coc-SA 10d, 1x/d, 20min	CPP re-test 7d, 1x/d, 15min 14d, 1x/d, 15min	**CPP**: 20min **Coc-prime**: 10 mg/kg		**CPP** **Coc-prime**	**CPP**: during EXT days **CPP**: saline prime

Behaviors in which adolescents are lower than adult are noted Adol <Adult and shaded in blue, equal Adol = Adult are shaded in white, and Adol > Adult are shaded in yellow. Adol, adolescent; Juv = juvenile, CPP = cocaine conditioned place preference, Coc-prime = cocaine prime, EXT = extinction, Pref test = preference test, N.S. = not specified, SD = sprague dawley.

### Expression of cocaine-CPP in adolescent and adult rats

The majority of cocaine CPP studies observed similar or higher CPP in adolescent rats compared to adults, with fewer studies showing lower CPP in adolescents. (Campbell et al., 2000; Badanich et al., 2006; Zakharova et al., 2009a,b). Specifically, at a conditioning dose of 5 mg/kg, late adolescent rats (corresponding to post-pubertal age) had similar cocaine CPP as their adult counterparts (Badanich et al., 2006). Another study also found that adolescent and adult rats (male and female combined) had similar cocaine CPP (Campbell et al., 2000). A subsequent study determined that female adolescent and adult rats had similar CPP, but that male adolescents displayed higher CPP than adults (Zakharova et al., 2009a,b).

At conditioning doses of 7.5–10 mg/kg, adolescent and adult rats exhibited similar or higher cocaine CPP (Campbell et al., 2000; Brenhouse et al., 2008; Brenhouse and Andersen, 2008). One study found that adolescent and adult rats (male and female combined) had similar CPP at 10 mg/kg (Campbell et al., 2000). Another set of studies found that adolescent male rats displayed higher CPP at doses of 7.5 and 10 mg/kg- only adolescent rats formed CPP at 7.5 mg/kg (Zakharova et al., 2009b), and adolescent rats displayed higher CPP compared to adult and juvenile groups when conditioned at 10 mg/kg (Brenhouse et al., 2008; Brenhouse and Andersen, 2008). A third set of studies found that both female and male adolescent rats were less sensitive to cocaine CPP than their adult counterparts (Zakharova et al., 2009a,b). Variables that could have contributed to different observations in adolescent sensitivity to CPP at 10 mg/kg include: (1) the age range of adult comparison groups- adolescents showing more CPP than adults at P103–105 age and less CPP when compared to adult at P66–70 age, (2) methodology used calculate cocaine CPP- use of the difference in time spent between saline and cocaine sides vs. the difference in time spent in the cocaine-paired side pre- vs. post-conditioning (Brenhouse et al., 2008; Zakharova et al., 2009a), and (3) results that combined vs. separated male and female behavioral data (Campbell et al., 2000; Zakharova et al., 2009b).

At higher conditioning doses of 20–40 mg/kg, adolescent and adult rats displayed similar or higher cocaine CPP. Adolescent rats had similar or higher cocaine CPP compared to juvenile or adults at 20 mg/kg; however, all three age groups had similar cocaine CPP at 40 mg/kg (Brenhouse et al., 2008). An additional study that matched the dose of cocaine used for CPP training to a separate group trained to self-administer cocaine (7.6–16.5 mg/kg across days) found similar levels of CPP between adult and adolescent rats (Guerin et al., 2021).

Several studies examined differences in cocaine-CPP between juvenile, adolescent, and adult rats; however, we only summarized findings that directly compared adolescent and adult age groups. One study found that juvenile rats showed less CPP than their adult counterparts (age range not specified) at a conditioning dose of 5 mg/kg (Hollis et al., 2012). Another study found that early adolescents (corresponding to pre-pubertal age) had higher CPP at 5 mg/kg when compared to late adolescent or adult rats (Badanich et al., 2006). We refer the reader to seminal papers that compared CPP in juvenile rodents that were not included in the current CPP summary table (Laviola et al., 1992; Pruitt et al., 1995; Bolanos et al., 1996; Brenhouse et al., 2015).

### Extinction of cocaine-CPP in adolescent and adult rats

To examine age-dependent differences in the strength of cocaine CPP, several studies incorporated extinction training sessions after the initial CPP test (Brenhouse and Andersen, 2008; Brenhouse et al., 2010; Guerin et al., 2021). During CPP re-tests, the time spent in each compartment was recorded and tests were repeated (daily over 7–14 days) until rats spent significantly less time in the cocaine-paired compartment compared to the initial test day (Brenhouse and Andersen, 2008). One of the first studies to evaluate extinction of cocaine CPP during adolescence found similar levels of CPP between age groups trained at 10 or 20 mg/kg cocaine, but that adolescent rats required more training sessions to reach extinction criteria (Brenhouse and Andersen, 2008). When a passive extinction strategy was used, adolescent rats also required more time than adults to reach extinction criteria; however when an explicit extinction strategy was employed, both age groups reduced CPP to a similar extent (Brenhouse et al., 2010). Consistent with these findings, a subsequent study found that adolescent and adult rats had similar CPP at cocaine doses matched to a separate cohort that received cocaine-SA training (7.6–16.5 mg/kg across days), but that adolescent rats failed to extinguish cocaine CPP when given twice as many extinction sessions as adults (Guerin et al., 2021).

### Operant cocaine self-administration (cocaine-SA) in adolescent and adult rats

Adult rodent models of cocaine-SA and relapse have been used to examine volitional drug-taking and -seeking behaviors (Venniro et al., 2016; Khoo et al., 2017; Feltenstein et al., 2021). In general, rats receive all behavioral training in operant boxes. During cocaine-SA, rats learn that active lever presses (or nosepokes) result in intravenous (i.v.) cocaine infusions, whereas inactive presses result in no consequences. Cocaine infusions are delivered on a range of fixed ratio (FR) schedules of reinforcement and length of training sessions can range between 1.5-6 h. Cocaine infusions are paired with discrete cues- tone + light complex, or passive, background stimuli- distinct olfactory, visual, auditory and tactile elements (Fuchs et al., 2008; Perry et al., 2014). After cocaine-SA training, rats undergo 1) 1-60 days of forced abstinence in the home cage, 2) extinction training in the same operant box where responses result in no cocaine or cue presentations, or 3) extinction in a second, distinct context where responses result in no cocaine. During reinstatement testing, rats are re-exposed to cocaine-associated contexts or cues and the number of responses on the previously reinforced lever is measured. An increase in responses after extinction or 1 day of abstinence is a measure of cocaine-seeking behavior, with significant higher responses (i.e., time-dependent increase) after 15–60 days of abstinence considered a measure of incubation of cocaine-seeking (i.e., incubation of craving) (Grimm et al., 2001; Li et al., 2016). During reinstatement tests, the impact of stress (footshock, yohimbine, or corticosterone) or cocaine priming (5–10 mg/kg) on cocaine-seeking can be assessed. A large body of work using adult rodent models of cocaine-SA has demonstrated that cues, context, stress and cocaine priming elicit cocaine-seeking behavior with cocaine-associated cues more likely to elicit incubation of craving (Tran-Nguyen et al., 1998; Grimm et al., 2001; Lu et al., 2004; Venniro et al., 2021). The adult preclinical literature on relapse and abstinence provide a solid foundation on which to compare adolescent studies.

### Cocaine-SA in adolescent and adult rats

**Figure 2:**
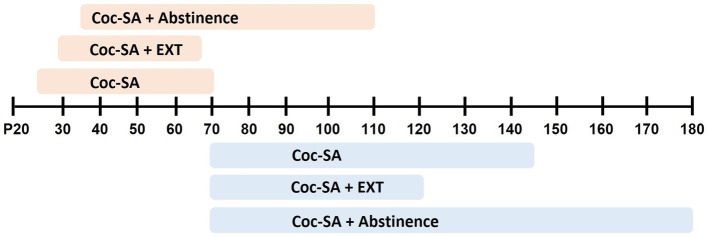
Summary of age range in experiments that employed cocaine self-administration (Coc-SA), Coc-SA and extinction (Coc-SA + EXT), or Coc-SA and drug-free abstinence period (Coc-SA + Abstinence). Shaded boxes indicate the length of behavior across postnatal days (P) for adolescent (orange) or adult (blue) rats.

In general, studies that examined age-dependent differences in cocaine-taking and -seeking behaviors started SA training in post-pubertal adolescents (range: P34–P44) and adults (range: P69–P97) (Figure 2). Studies in the current review used 1.5-3h short access (ShA) procedures (Belluzzi et al., 2005; Frantz et al., 2007; Kantak et al., 2007; Li and Frantz, 2009, 2017; Wong et al., 2013; Wong and Marinelli, 2016; Zbukvic et al., 2016; Li et al., 2018), 6h long-access (LgA) procedures (Wong et al., 2013; Madsen et al., 2017), or a combination of initial LgA training with ShA maintenance periods (Anker and Carroll, 2010; Holtz and Carroll, 2015). A smaller set of studies used intermittent training schedules that alternate between cocaine available vs. non-available (45 vs. 15 min) periods (Anker et al., 2011; Schramm-Sapyta et al., 2011). A last set of studies conduced cocaine-SA (2h ShA) in an an environmental context in the absence of explicit-paired cue (Cho et al., 2020; Guerin et al., 2021; Olekanma et al., 2023) (Table 2).

**Table 2:** Summary of key studies that compared adolescent and adult cocaine self-administration and cue-induced reinstatement (Cue cocaine-SA) or contextual reinstatement (Contextual cocaine-SA).

Cue Cocaine-SA										
Author	Rat Strain, Sex & Housing	Behavioral Range		Cocaine-SA Training	EXT Training	Drug-Free Period	Test	Adol < Adult	Adol = Adult	Adol > Adult
		Adol	Adult							
Belluzzi et al. (2005)	SD, ♂ Single	P27 – 31 (juv) P37 - 41	P90 - 95	250 μg/mL, 2d 750 μg/mL, 3d 5d, 3h/d, FR1	N/A	N/A	N/A		**SA response**: 5d total	
Frantz et al. (2007)	Wistar, ♂ Grouped	P38 - 51	P76 - 89	0.37, 0.74 mg/kg 14d, 2h/d, FR1	N/A	N/A	N/A		**SA response** **Total intake**: 0.37, 0.74 mg/kg	**SA response**: inactive 0.37 mg/kg
Kantak et al. (2007)	Wistar, ♂ ♀ Single	P37 - 49 dose resp: P50 - 57	P72 - 84 dose resp: P96 - 117	1.0 mg/kg initial 2h x 5 sess, FR1, FR5 0.1 - 3.0 mg/kg doses	N/A	N/A	N/A	**Intake**: 3.0 mg/kg ♂	**SA response** **Infusions**: 1.0 mg/kg	
Anker et al. (2011)	Wistar, ♂ ♀ Bred in house Housing N.S.	P26 - 50 ♀ P26 - 50 ♂	P93 - 143 ♀ P93 - 124 ♂	0.4 mg/kg, FR1 Avail: 45min x 3 Non-avail: 15min x 2	N/A	N/A	N/A			**Response**: ♂ ♀ Avail & Non-Avail **Intake**: ♂ ♀
Wong et al. (2013)	SD, ♂ Grouped	P35 - 41/44 P42 - 48/51	P88 - 94/97	0 - 1.2 mg/kg ShA: 7 - 10d, 1.5h/d LgA: 10d, 6h/d FR1, PR	N/A	N/A	N/A		**Infusions: ShA** 0.075, 1.2 mg/kg	**SA acquisition** **Infusions, PR**: 1.2 mg/kg **ShA**: 0.15, 0.3, 0.6 mg/kg **LgA**: 0.6 mg/kg, escalate
Holtz and Carroll (2015)	Wistar, ♀ Bred in house Paired	P25 - 70	P92 - 137	0.4 mg/kg 6h/d, FR1 10d, 2h/d x 2	N/A	N/A	N/A		**Infusions**	**SA response**: with histamine
Anker and Carroll (2010)	Wistar, ♂ Bred in house Housing N.S.	P27/29 - 58	P93/103 - 120	0.4 mg/kg 10d, 6h/d, FR1 5d, 2h/d x 2, FR1	2d, 2h/d 10d minimum (20 sess)	N/A	Cue Rein + Coc-prime Stress-prime	**Cue Rein**	**SA response** **Coc-prime**: 5 mg/kg	**Infusions**: over 5d **EXT response**: over 10d **Coc-prime**: 10, 15 mg/kg **Stress-prime**: Yohimbine
Zbukvic et al. (2016)	SD, ♂ Bred in house	P34 - 54	P69 - 89	0.3 mg/kg 2h/d 5 - 7d FR1, 5d FR3	EXT = 7d, 1h/d Cue EXT = 1d	N/A	Cue Rein +/- Cue EXT		**SA, PR response** **Infusions** **EXT response**	**Cue Rein** + Cue EXT
Schramm-Sapyta et al. (2011)	CD ♂ Single at train	6/10 - 12wk	11/15 - 17 wk	0.8 mg/kg 20d, 3h/d, FR1 Avail: 40min x 3 Non-avail: 15min x 2	Saline 20d max < 20% of Coc-SA	5d	Cue Rein		**Infusions**: 4 - 20d **Response**: Avail & Non-Avail **Cue Rein**	**Infusions**: 1 - 3d
Wong and Marinelli (2016)	SD, ♂ Grouped	P41/43 - 90	P88 - 140	0.6 or 1.2 mg/kg 10d, 2h/d, FR1 12d PR	1h, before test 21d, 1.5 h/d	40d or N/A	Cue Rein + Stress-prime		**Infusions**: 1.2 mg/kg **EXT response** **PR + EFS**: 1.2 mg/kg **Cue Rein**	**Infusions**: 0.6 mg/kg **Stress-prime**: Yohimbine Cort, EFS: 0.6, 1.2 mg/kg
Madsen et al. (2017)	SD, ♂ Bred in house Single	P35 - 45: 1d P35 - 75: 30d	P70 - 80: 1d P70 - 110: 30d	0.3 mg/kg 10d, 6h/d 5d FR1, 5d FR3	Cue EXT, day 7	1, 30d	Cue Rein after Cue EXT	**Discrimination**: Levers	**SA, EXT response** **Infusions, Intake** **Cue Rein**: 1, 30d +/- Cue EXT	
Li and Frantz (2009)	Wistar, ♂ Grouped	P35 - 49: 1d P35 - 63: 14d P35 -79: 30d P35 - 109: 60d	P97 - 110: 1d P97 - 124: 14d P97 - 140: 30d P97 - 171: 60d	0.36 mg/kg 14d, 2h/d, FR1	1d, 1h x 6 Before test	1, 14, 30, 60d Group house	Cue-Rein + Coc-prime	**EXT**: total 6h **Cue Rein**: 30, 60d	**SA response** **Infusions** **Cue Rein**: 1, 14d **Coc-prime**: 30d	
Li and Frantz (2017)	Wistar, ♂ Paired until drug-free	P35 - 107	P83/95 - 155/167	0.36 mg/kg 12d, 2h/d, FR1	1d, 1h x 6 Before test	60d Variable house	Cue Rein + Coc-prime	**EXT**: sess 1-2 **Coc-prime** **Cue Rein**: Standard, Impov	**SA response** **Infusions** **Cue Rein**: Enriched	
Li et al. (2018)	Wistar, ♂	P35 - 48: 1d P35 - 108: 60d	P83/95 - 96/108: 1d P83/95 - 156/186: 60d	0.36 mg/kg 12d, 2h/d, FR1	1d, 1h x 6 Before test	1, 60d Standard house	Cue Rein	**Cue Rein**: 1, 60d	**SA response** **Infusions** **EXT response** **Cue Rein**: 1d < 60d	
Contextual Cocaine-SA										
Guerin et al. (2021)	SD, ♂ Bred in house	P35 - 53/56	P70 - 88/91	0.3 mg/kg 10d, 2h/d 5d FR1, 5d FR3, PR	7d, 30min/d	N/A	Context Rein + Coc-prime	**Coc-prime**	**SA response** **Infusions** **EXT response** **PR response**	
Cho et al. (2020)	SD, ♂ Single or Paired	Non Abrv: P44/53 - 62 Abrv: P44/48-51	P74/83 - 92 P74/78 - 81	0.5 mg/kg 10d, 2h/d x 1 5d, 2h/d x 2 Context A	8d, 1h/d x 1 2d, 1h/d x 4 Context B	N/A	Context Rein		**SA response** **Adj Intake** **EXT response** **Context Rein**	
Olekanma et al. (2023)	SD, ♂ Single	P38/42 - 47: 1d P38/42 - 61: 15d	P70/74 -79: 1d P70/74 - 93: 15d	0.5 mg/kg 5d, 2h/d x 2 Context A	4d, 2h/d x 2 Context B	1, 15d	Context Rein		**SA response** **Adj Intake** **EXT response**	**Context Rein**: 1d < 15d, Adol
Herrera Charpentier et al. (2023)	SD, ♂ Single	P38/42 - 51	P70/74 - 82	0.5 mg/kg 5d, 2h/d x 2 Context A	4d, 2h/d x 2 Context B	N/A	Context Rein		**SA response** **Infusions** **EXT, MR response** **Context Rein**	

Behavioral phases in which adolescent are lower than adult are noted Adol < Adult and shaded in blue, Adol = Adult are shaded in white, and Adol > Adult are shaded in yellow. Adol, adolescent; Cocaine-SA, cocaine self-administration; EXT, extinction; Rein, reinstatement; PR, progressive ratio; MR, memory reactivation; N.S., not specified; SD, sprague dawley.

In general, studies that trained rats under ShA conditions with discrete cues observed that adolescent and adult rats had similar cocaine intake, infusions and active lever responses at doses of 0.3, 0.4, and 1.0 mg/kg (Belluzzi et al., 2005; Frantz et al., 2007; Kantak et al., 2007; Li and Frantz, 2009, 2017; Zbukvic et al., 2016). A study that conducted detailed dose–response curves under ShA conditions, found that adolescent and adult rats had similar intake at low and high doses of cocaine- 0.075 and 1.2 mg/kg. However, adolescents had faster acquisition of cocaine-SA, higher intake at moderate doses of 0.15, 0.3, and 0.6 mg/kg, and higher breakpoints on progressive ratio (PR) schedule of responding compared to adults (Wong et al., 2013). An additional study found that adolescent and adult rats trained under ShA conditions at 1.0 mg/kg had similar intake and PR responding, but that adolescents took less cocaine than their adult counterparts at 3.0 mg/kg (Kantak et al., 2007).

Under LgA conditions, adolescent rats self-administered more cocaine infusions than adults at a dose of 0.6 mg/kg (Wong et al., 2013) but had similar active responses and infusions at a dose of 0.3 mg/kg (Madsen et al., 2017). Adolescent rats that received a combination of LgA and ShA training with 0.4 mg/kg cocaine, had equal or higher active responses and infusions than their adult counterparts (Anker and Carroll, 2010; Holtz and Carroll, 2015). Two studies that used intermittent access schedules found that at 0.4 mg/kg, adolescent male and female rats had higher overall cocaine intake and responded more than adults during available and non-available periods (Anker et al., 2011). At higher doses of 0.8 mg/kg cocaine, adolescents also had higher infusions than adults on training days 1–3, but similar infusions on training days 4–20, with equal responses during available and non-available periods throughout the 20 training days (Schramm-Sapyta et al., 2011). A final set of studies trained rats to self-administer cocaine at 0.3 or 0.6 mg/kg in a distinct environmental context and found that both age groups had similar responses and cocaine intake in the absence of explicit cocaine-paired cues (Cho et al., 2020; Guerin et al., 2021; Olekanma et al., 2023).

### Extinction and abstinence in adolescent and adult rats

To examine age-dependent differences in cocaine-seeking or incubation of cocaine-seeking behavior, studies employed various extinction or abstinence procedures: daily extinction training over 7-21 days (Anker and Carroll, 2010; Schramm-Sapyta et al., 2011; Wong and Marinelli, 2016; Zbukvic et al., 2016; Guerin et al., 2021), 7 days of extinction followed by passive presentation of cocaine-paired cues (Cue-EXT), Cue-EXT on day 7 of a 30-day abstinence period (Zbukvic et al., 2016; Madsen et al., 2017) or 1-60 days of abstinence followed by extinction training before reinstatement (Li and Frantz, 2009, 2017; Wong and Marinelli, 2016; Li et al., 2018). Studies that employed cocaine-SA (ShA, 0.3, 0.6 mg/kg) and extinction training found that adolescent and adult rats had similar active responses across 7–21 daily extinction sessions (Wong and Marinelli, 2016; Zbukvic et al., 2016), while studies that used a combination of LgA and ShA training (LgA + ShA) found that adolescent rats had higher extinction responding over a total of 10 days (Anker and Carroll, 2010).

Investigations that incorporated 1–60 days of abstinence, followed by extinction training before reinstatement tests, found that after 30 days of abstinence, both age groups had similar extinction responding across 1 h sessions, but that adolescents had less extinction responses than adults when responses were totaled across all sessions. In a subsequent study, the investigators also found that adolescent rats had lower extinction responses during the 1^st^ two extinction sessions, compared to adults (Li and Frantz, 2009, 2017; Li et al., 2018). A final set of studies utilized passive presentations of a previous cocaine-paired cue (Cue-EXT) on subsequent cocaine-seeking, with or without periods of abstinence. In one of these studies, the investigators found that both age groups responded similarly on daily extinction training that occurred before Cue-EXT (Zbukvic et al., 2016).

### Reinstatement of cocaine-seeking in adolescent and adult rats

To understand the impact of adolescent vs. adult cocaine exposure on cocaine-seeking behavior, several studies examined cocaine-seeking elicited by cocaine-paired cues or re-exposure to a cocaine-paired context (Li and Frantz, 2009, 2017; Anker and Carroll, 2010; Schramm-Sapyta et al., 2011; Wong and Marinelli, 2016; Zbukvic et al., 2016; Madsen et al., 2017; Li et al., 2018; Cho et al., 2020; Guerin et al., 2021; Olekanma et al., 2023). In general, rodents that self-administered cocaine during adolescence displayed similar or reduced cue-induced cocaine-seeking when tested immediately after extinction training or after 1 day of abstinence (Li and Frantz, 2009; Schramm-Sapyta et al., 2011; Wong and Marinelli, 2016; Zbukvic et al., 2016). One study exposed rats to passive presentations of cocaine-paired cues (Cue-EXT) after extinction training and found that the adolescent cocaine-exposed group had higher cue-induced reinstatement than adults, suggesting that adolescent rats are less responsive to the Cue-EXT strategy. A set of studies that examined context-induced reinstatement also found that adolescent cocaine-exposed rats had similar levels of cocaine-seeking as adults (Cho et al., 2020; Olekanma et al., 2023).

A subset of the above reinstatement studies incorporated abstinence periods between cocaine-SA and testing to examine age-dependent differences in incubation of craving. Studies that used ShA or LgA conditions found that adolescent cocaine-exposed rats had similar or lower cue-induced cocaine-seeking after 30 or 60 days of abstinence compared to adults (Li and Frantz, 2009, 2017; Madsen et al., 2017; Li et al., 2018). Specifically, one study found that both age groups had higher responses after 60 days of abstinence compared to 1 day, but that overall, adolescents reinstated at lower magnitudes at both timepoints compared to adults. A second study found that adolescent cocaine-exposed rats had lower levels of cue-induced reinstatement after 30 days of abstinence (Li and Frantz, 2009). Another study found that adolescent and adult rats that received Cue-EXT on abstinence day 7, had similar cue-induced reinstatement when tested on abstinence day 30. Taken together with a previous study that found adolescents were less sensitive to Cue-EXT when tested without abstinence, these data suggest that adolescents may be less responsive to cue exposure therapies that are typically used in adult populations (Zbukvic et al., 2016; Madsen et al., 2017). In regard to contextual reinstatement, a study found that adolescent exposed rats had similar responses after 1 day of abstinence, but that only adolescent rats displayed higher responding after 15 days of abstinence (Cho et al., 2020; Olekanma et al., 2023). A timecourse analysis during the contextual reinstatement test showed that adolescent cocaine-exposed rats had higher responding throughout the test, suggesting a potential resistance to extinction within the cocaine-paired context (Olekanma et al., 2023).

Finally, a separate set of studies examined whether cue-induced reinstatement was influenced by exposure to stress or cocaine-priming. In general, adolescent rats displayed similar or reduced cocaine-primed reinstatement at doses of 5 mg/kg when tested immediately after extinction or after 60 days of abstinence (Anker and Carroll, 2010; Li and Frantz, 2017). At a 10 mg/kg priming dose, adolescent rats displayed varying responses- one study found adolescents had lower cocaine-primed reinstatement when tested after extinction or after 60 days of abstinence (Li and Frantz, 2017; Guerin et al., 2021), but that both age groups had similar reinstatement when tested after 30 days of abstinence. A second study found that with priming doses of 10 and 15 mg/kg, adolescents had higher cocaine-seeking than adults when tested after extinction (Anker and Carroll, 2010). Variables that could have contributed to the different cocaine-primed results at 10 mg/kg include ShA vs. LgA + ShA cocaine procedures, extinction training that occurred after an abstinence period (6, 1 h sessions) vs. 7–20 daily extinction sessions (Anker and Carroll, 2010), and use of between-subjects vs. within-subjects testing design during reinstatement tests.

In regard to stress-induced reinstatement, studies have found that adolescent cocaine-exposed rats show heightened stress-induced reinstatement when compared to adults. The heightened sensitivity to stress was observed with ShA vs. LgA + ShA cocaine-SA procedures, extinction training that occurred after 40 days of abstinence vs. 7–21 daily extinction sessions, and with different stressors including yohimbine, corticosterone, or electric footshock (Anker and Carroll, 2010; Wong and Marinelli, 2016).

## Conclusions and future experimental considerations

Overall, preclinical studies that utilized cocaine-CPP and cocaine-SA procedures have increased our knowledge on the impact of adolescent cocaine exposure on cocaine reward, relapse and incubation of craving. Several studies showed that adolescent rats have similar cocaine CPP and SA behavioral profiles when compared to adults, however their responses to subsequent relapse triggers and extinction of cocaine associations are complex. Adolescent rats may initially have similar behavioral profiles as their adult counterparts, however dynamic changes that occur during the drug-free periods may not be revealed until later in life. Future avenues for research include investigating the complex relationship between cocaine exposure during adolescence and subsequent effects on learning. Seminal papers have shown that exposure to cocaine during adolescence can lead to changes in stimulus reward learning, reversal learning, reinforcement-learning trajectories, and habit-like behaviors (Kerstetter and Kantak, 2007; Harvey et al., 2009; Kantak et al., 2014; DePoy et al., 2016; Kantak, 2020; Moin Afshar et al., 2020; Villiamma et al., 2022). Few studies have investigated whether adolescent drug memories may be weakened to prevent subsequent craving and relapse in adulthood. A focus on reducing the reconsolidation of adolescent drug memories would be an interesting avenue to pursue given that adolescent rats are more resistant to extinction of cocaine CPP and operant behavior (Bender and Torregrossa, 2020; Herrera Charpentier et al., 2023). It is known that adolescents are particularly sensitive to the effects of peers and therefore future studies should be designed to examine the impact of adolescent social interactions to exacerbate or mitigate cocaine-SA, reward, relapse, and craving.

An additional avenue for future research should investigate the underlying circuit mechanisms that contribute to craving and relapse in adolescent cocaine-exposed rats. Findings from human literature suggest that prefrontal, limbic and reward regions important for flexible decision making, formation of drug-associations and reward undergo massive reorganization during adolescence (Chambers et al., 2003; Caballero et al., 2016). Key preclinical investigations have found evidence for age-dependent differences in the ventral tegmental area and prelimbic to accumbens circuits during cocaine CPP and operant SA (Brenhouse et al., 2008; Wong et al., 2013). Modulation of these circuits after periods of abstinence would be an important next step in understanding their role in adolescent craving and relapse.

## Author contributions

AA: Writing – original draft. CV: Writing – original draft. LV: Writing – review & editing. CR: Writing – review & editing.
